# Photo-fluorination of nanodiamonds catalyzing oxidative dehydrogenation reaction of ethylbenzene

**DOI:** 10.1038/s41467-021-26891-8

**Published:** 2021-11-11

**Authors:** Zhishan Luo, Qiang Wan, Zhiyang Yu, Sen Lin, Zailai Xie, Xinchen Wang

**Affiliations:** 1 State Key Laboratory of Photocatalysis on Energy and Environment, College of Chemistry, Fuzhou University, Fuzhou, Fujian 350108 People’s Republic of China; 2 College of Chemical Engineering, Fuzhou University, Fuzhou, Fujian 350108 People’s Republic of China

**Keywords:** Heterogeneous catalysis, Photocatalysis, Photocatalysis

## Abstract

Styrene is one of the most important industrial monomers and is traditionally synthesized via the dehydrogenation of ethylbenzene. Here, we report a photo-induced fluorination technique to generate an oxidative dehydrogenation catalyst through the controlled grafting of fluorine atoms on nanodiamonds. The obtained catalyst has a fabulous performance with ethylbenzene conversion reaching 70% as well as styrene yields of 63% and selectivity over 90% on a stream of 400 °C, which outperforms other equivalent benchmarks as well as the industrial K−Fe catalysts (with a styrene yield of 50% even at a much higher temperature of ca. 600 °C). Moreover, the yield of styrene remains above 50% after a 500 h test. Experimental characterizations and density functional theory calculations reveal that the fluorine functionalization not only promotes the conversion of *sp*^*3*^ to *sp*^*2*^ carbon to generate graphitic layers but also stimulates and increases the active sites (ketonic C=O). This photo-induced surface fluorination strategy facilitates innovative breakthroughs on the carbocatalysis for the oxidative dehydrogenation of other arenes.

## Abstract (web summary)

Styrene is one of the most important industrial monomers and is traditionally synthesized via the dehydrogenation of ethylbenzene. Here, the authors report a photo-induced fluorination technique to generate an oxidative dehydrogenation catalyst through the controlled grafting of fluorine atoms on nanodiamonds.

## Introduction

Styrene (ST) is one of the most widely used precursors of fine chemical synthesis, as well as for the preparation of plastics and rubbers, with a global production of *ca*. 30 million tons in 2018^1^. It is nowadays industrially produced by direct dehydrogenation of ethylbenzene (EB) at 550−650 °C, using K-Fe-based catalysts with a styrene yield of ca. 50%, and during this period, a large amount of steam must be applied simultaneously as a co-feed to alleviate catalyst coking^2^. This traditional process is typically restricted by thermodynamics and a large amount of energy dissipation^2^. To overcome these limitations, tremendous efforts have been devoted to developing advanced technologies for the production of styrenes^3^. Among these technologies, the oxidative dehydrogenation (ODH) of alkanes and arenes by carbon catalysis have been regarded as an effective and sustainable approach to tackling the barrier^4,5^. However, the disordered or amorphous carbon-based catalysts have shown a low activity, unsatisfactory selectivity, and/or poor stability. In pursuit of well-structured carbon-based catalysts, the rapid development of nanoscience and nanomaterials, with nanocarbons (fullerene, graphene, nanodiamonds, and nanotubes) as the flagship, has extended carbon-based catalysts^6^, and exhibits an excellent performance in the process of ODH reactions^7^.

Nanodiamond (ND) is one of the most important nanocarbon catalysts, with a unique *sp*^*3*^-hybridized structure, large surface-to-volume ratio, stable chemical properties, and good biocompatibility^8,9^. It has been intensively investigated and applied in many fields, including lubrication^10^, nanoscale magnetic sensors^11^, and biomedical applications^12^. The extraordinary prospect of NDs in ODH reactions has been appreciated due to its special *sp*^*3*^/*sp*^*2*^ core-shell structure. However, the *sp*^*3*^-carbon of ND would lead to C−C cleavage and benzene formation in the ODH of EB, and the agglomeration of ND by surface bonding force suppresses its catalytic activity^13^. In recent years, considerable research efforts have focused on improving the ODH performance based on ND catalysts by identifying and exposing active sites^14^, as well as nanostructuring^15^, surface engineering^16^, and hybridization^17^. Among these approaches, surface engineering seems to be an efficient, simple, and cost-effective strategy^18^. It is necessary to further explore the influence of surface-modified NDs on catalytic performance because a large percentage of carbon atoms, defect sites, and functional groups are located on the surface^19^. Therefore, the rational design and optimization of the special *sp*^*3*^/*sp*^*2*^ core-shell structure of ND have a great practical significance for ODH reactions.

The surface modification of ND has been studied during the past decade and fluorination has been regarded as an efficient way to modify and control the surface properties of ND^20^. So far, the diamond surface has been fluorinated only with extreme methods involving molecular F_2_, atomic F, XeF_2_, fluorine-containing plasmas, and X-ray irradiation^20–22^. However, each of these surface modification methods involves the handling of corrosive gases under harsh treatment conditions and causes serious pollution to the environment. In view of the green and sustainable chemistry, fluorination of ND by using a mild and simplified tactic is desired. Here, we report a photo-induced fluorination strategy to synthesize fluorine-functionalized nanodiamonds (F-ND). Compared to traditional carbon catalysts and the industrial K−Fe catalyst, the F-ND displays a much-enhanced ethylbenzene conversion and styrene selectivity at a relatively low temperature of 400 °C. The improved performance of ODH of EB can be ascribed to the increasing contents of graphitic carbon and the content of active sites (ketonic C=O) by fluorine incorporation. This work reveals an important strategy for a new avenue for fluorination and property modification of carbon-based catalysts and presents a wide range of possibilities for the further development of ODH reactions.

## Results

### Catalyst synthesis and characterization

**Fig. 1:**
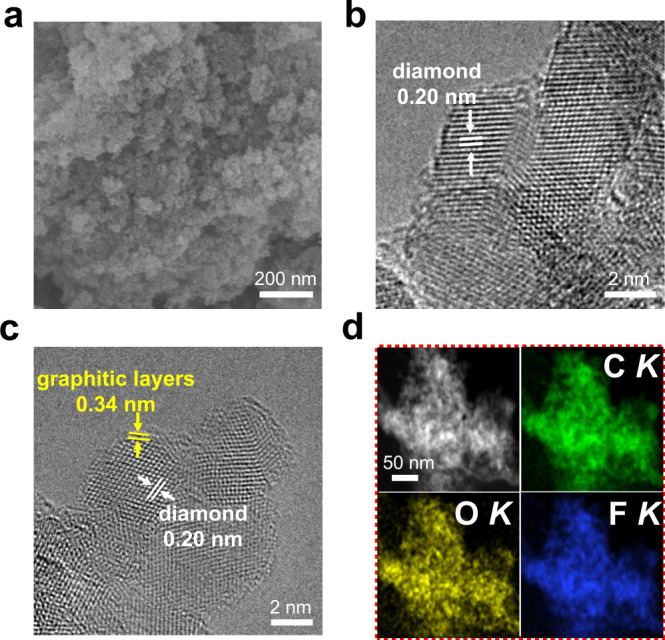
Microscopic characterization of ND and F-ND catalysts. **a** SEM image of F-ND catalysts. **b** TEM image of ND catalysts. **c** High-resolution TEM image, and **d** corresponding elemental maps of F-ND catalysts, showing the distributions of C *K* (green), O *K* (yellow), and F *K* (blue).

The details for the photo-fluorination of ND were described in the Methods section. Briefly, the ND nanoparticles were soaked in the C_4_F_9_I solution with stirring, followed by photoinduction with the Xeon lamp for few hours. The obtained catalyst was denoted as F-ND catalysts. A possible mechanism for the photo-fluorination of ND catalysts was proposed in Supplementary Fig. 1. After the treatment of fluorination, the texture of ND and F-ND catalysts were explored by scanning electron microscopy (SEM). A multitude of small balls can be observed from Fig. 1a together with Supplementary Fig. 2 for F-ND and ND catalysts, respectively. Further experiments of Cs-corrector transmission electron microscopy (Cs-TEM) were also performed. The ND catalyst was covered with a small amount of amorphous graphite (Fig. 1b) and a core-shell structure covered with few graphitic layers was formed after the fluorination strategy (Fig. 1c). Additionally, the interplanar spacings of 0.20 and 0.34 nm corresponding to the (111) and (002) crystallographic planes for the ND and graphite were also observed, respectively. The core-shell structures were further confirmed by the statistics of the particle size and the number of graphite layers for ND and F-ND catalysts, respectively (Supplementary Fig. 3). The F-ND catalysts exhibited an average particle size of 5.2 $\pm$ 0.4 nm and were covered with 2−4 graphitic layers, which were smaller than the ND catalysts (average particle sizes are 5.8 $\pm$ 0.4 nm). The high angle annular dark-field (HAADF) image and corresponding elemental maps of the F-ND showed that the catalyst has consisted of carbon, oxygen, and fluorine elements with a uniform distribution (Fig. 1d).

To further investigate the structure of the F-ND catalysts, the powder X-ray diffraction (XRD) pattern and the Raman spectra were collected (Supplementary Fig. 4a, b). A barely noticeable difference could be observed, indicating that the substrate of ND maintains a similar crystallization degree after fluorine doping. Notably, the peaks in the XRD spectra located at 43.7°, 75.3°, and 91.5° were ascribed to the (111), (220), and (311) diffraction planes of the diamond, respectively^23^. A weak broad peak located at 26.3° corresponds to the (002) diffraction of the graphite crystal plane, which was consistent with the Cs-TEM result^15^. The N_2_ adsorption−desorption isotherm curve of the F-ND with a specific surface of 275 m^2^ g^−1^ showed nearly the same absorbed volume as ND with 280 m^2^ g^−1^ (Supplementary Fig. 4c), suggesting that the photo-induced fluorination method did not change the surface area of ND catalysts. The fluorine signals were explored by the X-ray photoelectron spectroscopy (XPS), Fourier transform infrared (FT-IR) spectra, and ^19^F NMR spectra, as shown in the Supplementary Fig. 4d−f, respectively. The high-resolution XPS of F 1s for F-ND catalysts was fitted with two peaks located at 688.3 and 684.7 eV, which belonged to CF_2_ and CF, respectively^24^. FT-IR spectra also demonstrated that the existence of CF_2_ and CF structure in the F-ND catalysts with 1229 and 1422 ${{{{{{\rm{cm}}}}}}}^{-1}$ for CF_2_, and 1076−1179 ${{{{{{\rm{cm}}}}}}}^{-1}$ for CF. In addition, notable peaks at ca. 1784 and 1629 ${{{{{{\rm{cm}}}}}}}^{-1}$ were observed, which corresponded to the C=O and O−H modes^24,25^. In the ^19^F NMR spectrum, the peaks at −84 and −100 ppm corresponded to the CF_2_ sites, while the peaks at −121, −149, and −175 ppm belonged to the CF sites^24–26^. Therefore, we successfully synthesized two kinds of F-bonded nanodiamonds, and the main structure of the nanodiamond was maintained (only containing several layers of graphite on its surface).

### Catalytic results

**Fig. 2:**
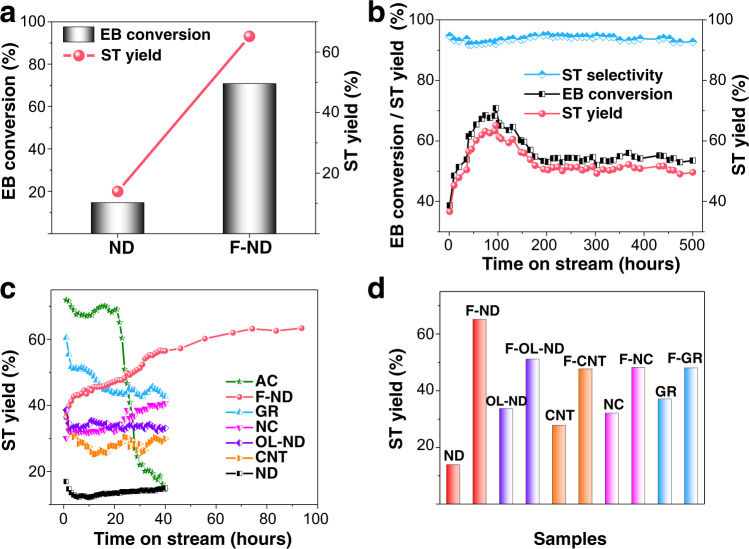
ODH activities of EB to ST for various carbon-based catalysts. **a** The performance of ND and F-ND catalysts for ODH reactions. **b** Stability of F-ND catalysts for ODH reactions over a 500 h test. **c** Stability of carbon-based catalysts for ODH reactions over 40 h test. **d** The optimized performance of fluorinated carbon-based catalysts for ODH reactions. Reaction conditions for all the experiments: 20 mg catalysts, 470 nmol mL^−1^ EB with N_2_ balance, O_2_: EB = 3:1, total flow rate = 10 mL·min^−1^, *T* = 400 °C. AC active carbons. GR graphene. NC nanocarbons. OL-ND onion-like nanodiamonds. CNT carbon nanotubes.

We conducted the reaction at 400 °C with an O_2_/ethylbenzene ratio of 3 to 1. The product mixture contained styrene, benzene, CO_2_, and residual reactants. The resulting carbon balance was 100 $\pm$ 5% (Supplementary Fig. 5). The highest catalytic performance of ND and F-ND catalysts for ODH reactions were shown in Fig. 2a. The F-ND catalysts delivered a high catalytic performance with 70.8% conversion of ethylbenzene and 65.2% yield of styrene, which was 4.8 times (14.6%) and 4.7 times (13.9%) higher than the pristine ND catalysts, respectively. The F-ND catalysts still maintained a styrene yield of 50% in the 500 h test, indicating its excellent stability (Fig. 2b). It was worth noting that a deactivation step was observed around 100 h may be due to the formation of graphitic carbon to amorphous on the surface of F-ND catalysts and the reduction of the content of active sites during the reaction, which causes a partial decrease in catalytic performance (Supplementary Fig. 6)^27^. Furthermore, a probe reaction of the dehydrogenation of 1,2,3,4-tetrahydroquinoline (THQ) reaction was carried out to characterize the dehydrogenation activity for ND and F-ND catalysts (Supplementary Fig. 7)^28^. The yield of quinoline for F-ND catalysts is also higher than ND catalysts, illustrating that F-ND catalysts have better dehydrogenation activity, indicating a broad application in dehydrogenation reaction fields^29^.

The thermal stability and oxidation resistance were identified by thermogravimetric analysis (TGA), as shown in Supplementary Fig. 8a. The 3 h fluorination seemed to be the best time to fabricate the catalysts (F-ND-3h) with the highest thermostability and oxidation resistance. In addition, the measurements of XRD, XPS, and Raman for F-ND catalysts before and after the ODH reactions for 100 h test in Supplementary Fig. 8b−d also showed no obvious changes except a few graphitization, which may be attributed to fluorine doping and the oxygen-containing functional groups bonded to the *sp*^2^ structure^30^.

The ODH reaction behaviours of various carbon-based catalysts were illustrated in Fig. 2c. The NDs, onion-like NDs (OL-ND), nanocarbons (NC), and carbon nanotubes (CNT) catalysts showed a low yield of styrene. Active carbons (AC) catalysts provided the highest initial activity, but the lowest stability was observed in this amorphous carbon material. In comparison, the graphene (GR) catalysts although obtained a better yield of styrene than F-ND catalysts at the beginning, but after 15 h of tests, the yield of GR catalysts decreased gradually. In addition, the performances of NC catalysts were growing after 40 h, and finally, after nearly 300 h of the test, the yield of styrene for NC catalysts stabilized at *ca*. 20% without increasing trend (Supplementary Fig. 9). Moreover, the same photo-induced etching technique was used for the fluorination of OL-ND, CNT, NC, and GR catalysts. All the fluorine-modified catalysts exhibited an enhanced performance of the ODH reactions (Fig. 2d and Supplementary Fig. 10), indicating the universality of this method. Furthermore, we also synthesized and evaluated the other element (B, P) modified ND catalysts and fluorine precursor (NH_4_HF_2_, HF) processed ND catalysts. Under the same conditions, the photo-induced synthesized F-ND catalysts were recorded with the best performance (Supplementary Fig. 11). In addition, compared with commercial K−Fe_2_O_3_ catalysts, the F-ND catalysts exhibited a higher styrene yield.

Further exploring the optimum synthetic conditions by adjusting the time and the concentration of the C_4_F_9_I solution for photo-induced fluorination. The best conditions of the fluorination time and the content of C_4_F_9_I were 3 h and 3 mL, respectively (Supplementary Fig. 12). In addition, the Arrhenius activation energies from the ODH reaction rates were obtained at different temperatures (Supplementary Fig. 13), and the reaction orders for ethylbenzene or O_2_ from the linear equation of ${{{{{\rm{In}}}}}}R\approx {{{{{\rm{m}}}}}}\,{{{{{\rm{In}}}}}}{Pi}$ (Supplementary Fig. 14 and Supplementary Table 1). These values were comparable to previously reported results^4,31^. We thus concluded that the kinetic data agreed well with the dual-site Langmuir−Hinshelwood model. Consequently, we introduced fluorine into the surface of ND catalysts and shown a dramatically enhanced performance for the oxidative dehydrogenation of ethylbenzene. We compared fluorine element with other elements (such as B and P), and the result was that the fluorine-modified ND showed a better property. In addition, different sources of fluorine from NH_4_HF_2_ and HF to etch the ND catalysts also proved to have low performances, indicating the photo-induced etching method by using the C_4_F_9_I was unique and better than other approaches. The data of DH and ODH of EB to ST for various catalysts were listed in Supplementary Table 2, and the F-ND catalysts maintained a better conversion and selectivity for the reactions.

### Active sites for catalytic oxidative dehydrogenation of ethylbenzene

**Fig. 3:**
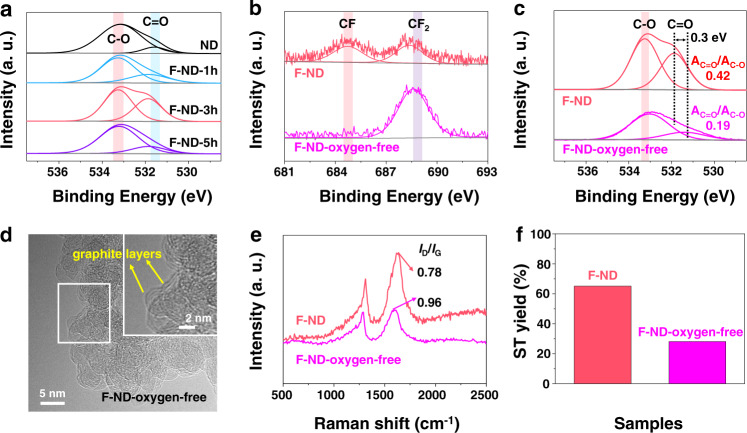
The role of oxygen during the photo-induced fluorination strategy. **a** High-resolution XPS O 1s spectra of ND and F-ND catalysts with different fluorination time. High-resolution XPS spectra of **b** F 1s and **c** O 1s for F-ND and F-ND-oxygen-free catalysts, respectively. **d** TEM images of F-ND-oxygen-free catalysts. **e** Raman spectra, and **f** the performance of ODH reactions for F-ND and F-ND-oxygen-free catalysts, respectively.

The TEM images confirm that the surface of F-ND was covered with a few graphitic layers, although the structure was not apparently changed, indicating that the photo-induced fluorination process in the presence of oxygen may produce some functional groups on the surface of ND catalysts. Previous reports have shown that the ketonic carbonyl groups (C=O) were the active sites for ODH reactions^14,17,32^. A significant change in the amounts of ketonic C=O and C−O groups could be observed after the photo-induced fluorination process (Fig. 3a). At 1 h, the area of the peak ratio of C=O groups and C−O groups (A_C=O_/A_C−O_) was calculated as 0.28 (Supplementary Table 3), which was higher than that of ND catalysts (0.11). When the fluorination time increased to 3 h, the value of A_C=O_/A_C−O_ increased to 0.42, implying more C=O groups were generated. Note that the peak shift of oxygen after fluorination was 0.3 eV resulting from the strong electronegativity of the F atom, reflecting the presence of fluorine^33^.

**Fig. 4:**
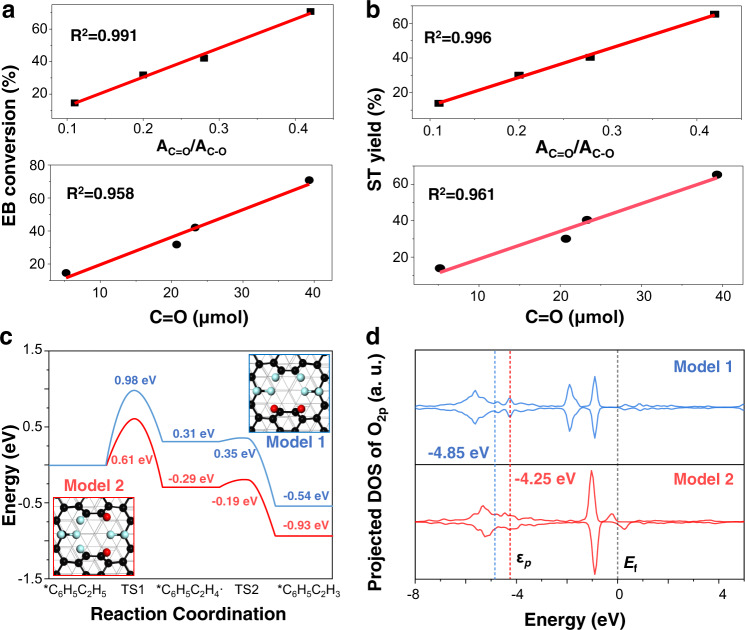
The origin of active centre and proposed reaction mechanism of EB dehydrogenation over F-ND catalysts. **a** Linear fitting of EB conversion of A_C=O_/A_C−O_ and mole of C=O, respectively. **b** Linear fitting of the ST yield of A_C=O_/A_C−O_ and mole of C=O, respectively. **c** DFT calculated reaction pathways of EB dehydrogenation on F-ND catalysts with two C=O groups without (Model 1, blue curve) and with (Model 2, red curve) an F atom located at an adjacent carbon atom to C=O. colour scheme: C in the outmost graphene surface, black; other C atoms, grey; O, red; F, light blue. **d** Projected density of states (PDOSs) of O_2*p*_ states in C=O groups without (upper panel) and with (lower panel) an F located at an adjacent carbon atom to C=O. The corresponding *p*-band centre was highlighted with the Fermi level (*E*_F_) set to zero. TS transition state.

To uncover the role of oxygen during the photo-induced fluorination strategy, a control experiment of photo-fluorination of ND catalysts in the absence of oxygen gas (F-ND-oxygen-free) was conducted (see the “Method” section for the detailed synthetic method of F-ND-oxygen-free catalysts). Figure 3b, c shows the XPS spectra of F 1s and O 1s for F-ND and F-ND-oxygen-free catalysts, respectively. The F-ND-oxygen-free catalysts only contain CF_2_ bonds with a low content of C=O bonds (the A_C=O_/A_C−O_ is calculated as 0.19) under the condition of the photo-fluorination process in the absence of oxygen. On the contrary, when oxygen participated in the procedure of photo-fluorination, not only a new CF bond was produced on the surface of F-ND catalysts, but also the content of the C=O bond was significantly increased (the A_C=O_/A_C−O_ is calculated as 0.42). In addition, several graphitic layers covering the surface of F-ND-oxygen-free catalysts were confirmed by the result of TEM images (Fig. 3d), and the intensity ratio of the I_D_/I_G_ from the Raman spectrum decreased from 0.96 to 0.78, indicating that the graphitization degree of the F-ND catalyst was higher than that of the F-ND oxygen-free catalyst (Fig. 3e)^27^. Catalytic test also indicated that a significant decrease in the activity of F-ND-oxygen-free catalysts was observed in Fig. 3f when compared with F-ND catalysts, illustrating the importance of oxygen in the photo-fluorination process. Furthermore, we also correlated the values of EB conversion and ST yield to the values of A_C=O_/A_C−O_. Figure 4a, b showed that the A_C=O_/A_C−O_ presented a positive linear relationship with the EB conversion and ST yield, the obtained correlation coefficient (*R*^2^) was as high as 0.991 and 0.996, respectively. This result suggested that the ketonic C=O groups made a substantial contribution to the performance of ODH for the ethylbenzene reactions.

To further clarified the role of the ketonic C=O groups, chemical titration of oxygen functional groups was carried out^14^, and the hydroxylamine hydrochloride was used for the determination of ketonic C=O groups, which was a reliable and classical quantitative method^34^. When the photo-induced fluorination time was 3 h, the concentration of the C=O groups reaches the maximum value (Supplementary Table 4). By the correlation analysis, the obtained value of *R*^2^ for the mole content of the C=O group was 0.958 and 0.961 for EB conversion and ST yield, respectively (Fig. 4a, b). Evidently, the above results further illustrated the ketonic C=O groups could be the active centres for the ODH reactions and the surface oxygen-containing groups could be effectively tailored by mild photo-induced fluorination treatment.

### Reaction mechanism studied by density functional theory calculations (DFT)

The DFT calculations have been performed to understand the reaction mechanism of oxidative dehydrogenation and the nature of active sites on the F-ND catalysts (the computational methods were detailed in the supplementary materials). The F-ND catalysts model was based on a diamond slab covered by two graphene monolayers (Supplementary Fig. 15), in which the layer spacing (0.33 nm) was consistent with our experimental value (0.34 nm, Fig. 1c). To simulate the ketonic carbonyl groups (C=O) observed in the experiment, two possible models with oxygen atoms introduced onto the carbon vacancies after fluorination was constructed (Model 1 and Model 2 without and with an F located at an adjacent carbon atom to C=O, respectively, Fig. 4c).

Two dehydrogenation steps of EB were considered in our calculations: the dehydrogenation of α-H of EB to produce C_6_H_5_C_2_H_4_ intermediate, followed by its dehydrogenation to generate C_6_H_5_C_2_H_3_ products. In Model 1, the calculated energy barriers for these two dehydrogenation steps were 0.98 and 0.04 eV, respectively, indicating the former one as the rate-determining step (Fig. 4c). While on Model 2, the dehydrogenation of EB became much easier with the reaction barrier as low as 0.61 eV. Obviously, the catalytic activity depends on the location of F groups directly. The configurations along the reaction pathway can be seen in Supplementary Figs. 16 and 17. The calculated projected density of states (PDOSs) of the O 2*p* orbitals in C=O (Fig. 4d) illuminated that the existence of fluorine adjacent to C=O leads to the upshift of the *p* states toward the Fermi level (*E*_F_) with the *p*-band centre significantly changed from −4.85 in Model 1 to −4.25 eV in Model 2. This result suggested the C=O group becomes more active for catalyzing the dehydrogenation of EB when fluorine was located at the adjacent carbon atom. In addition, the effect of C=O concentrations on the catalytic activity of dehydrogenation of EB was examined. The calculation results showed that the energy barrier decreases with the increase of C=O concentration (Supplementary Figs. 18 and 19), consistent with the trend observed in our experiment (Fig. 4c, d). Moreover, to explain differences between F-ND and F-GR catalysts, we performed additional DFT calculations by removing the nanodiamonds from the F-ND model (Supplementary Fig. 18, N_C=O_ = 1). According to the high-resolution XPS spectra of F 1s that both CF_2_ and CF bonds were observed on F-ND catalysts while only CF_2_ bond was detected on F-GR catalysts (Supplementary Fig. 20a). The calculated energy barriers of the dehydrogenation of EB reaction on F-ND and F-GR catalysts were 1.24 and 1.44 eV, respectively, indicating the different behaviour between F-ND and F-GR catalysts (Supplementary Fig. 20b, c).

### The chemical role of fluorine

It is meaningful to understand the chemical role for F in the reactions, especially whether it is in close proximity to C=O. Therefore, we focus on elaborating the role of F in our study with a series of additional measurements (Fig. 3 and Supplementary Fig. 21).

As we all know, the element of F has a strong electron-withdrawing ability^35^, which can significantly change the character of adjacent bonds. Therefore, high-resolution XPS spectra of F 1s and O 1s for F-ND and F-ND-oxygen-free catalysts are carried out in Fig. 3b, c, respectively. In the process of photo-fluorination under the condition of oxygen, the F-ND catalysts contain two types of CF and CF_2_ bonds (Fig. 3b). More importantly, the O 1s peak slightly shifts 0.3 eV after fluorination (Fig. 3c), which is due to the strong electronegativity of the F atom, reflecting the presence of fluorine is close to the C=O bond (the distance is less than three C−C bonds apart^36,37^). Inversely, the F-ND-oxygen-free catalyst only contains CF_2_ bonds, and no obvious shift is observed at the O 1s peak (Fig. 3b, c). This indicates that the electron-withdrawing ability of the C−F bond acting on the C=O bond is weakened, as a result of the long distance between C−F and C=O bonds more than three C−C bonds apart^36,37^. Furthermore, the area of the peak ratio of C=O groups and C−O groups (A_C=O_/A_C−O_) is calculated as 0.42 and 0.19 for F-ND and F-ND-oxygen-free catalysts (Fig. 3c), respectively. This result shows that the active sites of C=O groups on the surface of F-ND catalysts are increased after the C−F bonding on the surface of catalysts during the photo-fluorination process in the presence of oxygen.

We can also get an insight into the role of the F element from the HRTEM image, Raman spectrum, and the performance of ODH reactions of the F-ND and F-ND-oxygen-free catalysts. Figure 3d shows that a few graphite layers are formed after photo-fluorination treatment, and the *I*_D_/*I*_G_ decreases from 0.96 to 0.78 (Fig. 3e), displaying the fine graphitic structure of F-ND catalysts after functionalization of fluorine^27^. More importantly, a significant decrease in the activity of F-ND-oxygen-free catalysts is observed in Fig. 3f when compared with F-ND catalysts, indicating the role of F (with the ability of electron-withdrawing and increased active sites) are beneficial to the ODH reactions.

In addition, we constructed two model structures with different distances between the C−F and C=O bonds (Supplementary Fig. 21, Model 3: the C−F bonds are adjacent to C=O bonds, Model 4: the bonds of C−F and C=O are separated by three C−C bonds) for additional DFT calculations. The calculated projected density of states of the O_2*p*_ orbitals in two model structures illuminated that the existence of fluorine adjacent to C=O leads to the upshift of the *p*-band centre from −4.29 in Model 4 to −4.19 eV in Model 3, suggesting the C=O group become more active in catalyzing the ODH reactions when fluorine is located at the adjacent carbon atom, which is consistent with the performance of ODH reactions.

Finally, these results show that a convenient and environmentally friendly strategy for photo-fluorination of ND catalysts can form CF and CF_2_ bonds on the surface of F-ND catalysts with strong electron-withdrawing ability. This photo-fluorination process increases the content of C=O active sites and makes the surface of F-ND catalysts forming a fine graphitic structure, which can facilitate the performance of ODH reaction.

## Discussion

In summary, we have demonstrated a photo-induced process to fabricate the fluorine-modified ND for the ODH of ethylbenzene. The fluorine modification ND can stabilize the active site (ketonic C=O group) and activate the surface graphitic layers, leading to a conversion of ethylbenzene as high as 70% at the temperature of 400 °C, with a styrene selectivity over 90%. The long-term stability testing indicates that there is no significant deactivation even after reaction for 500 h. The DFT calculations further reveal that the fluorination of ND reduces the reaction energy of the ODH process, which improves the adsorption ability to ethylbenzene and reduces the activation energy for the ODH reaction. Overall, this technique is equally applicable to fluorination of other carbon-based catalysts, which may open a new possibility for fundamental studies of ODH of ethylbenzene. It is expected such a photo-induced surface fluorination strategy can guide the design of carbon-based industrial catalysts for ODH of light hydrocarbons into high value-added chemicals.

## Methods

### Synthesis

The F-ND catalysts were synthesized by the photo-induced etching method. Typically, 40 mg nanodiamond (ND, ≥ 97 %, Nanjing XFNANO Materials Tech Co., Ltd) and 3 mL nonafluoro-1-iodobutane (C_4_F_9_I, ≥ 98%, Aladdin) were mixed into the round-bottomed flask and the temperature of the reaction solution was maintained at 30 °C controlled by a flow of warming water during the reaction. Then the entire system was vigorously stirred and illuminated for 3 h under a 300 W xenon lamp (CX-05E, AC input 200−240 V, Eagle engineering CO., Ltd) with an irradiation power density of 649 ${{{{{\rm{mW}}}}}}/{{{{{{\rm{cm}}}}}}}^{2}$ (Supplementary Fig. 22). After the reaction, the sample was washed several times with purified water and collected by centrifugation. Finally, the sample was drying at 80 °C ovens overnight. The photo-fluorination of other carbon-based catalysts, including onion-like NDs (OL-ND), carbon nanotubes (CNT), nanocarbons (NC), and graphene (GR) catalysts, were synthesized by the same steps described in the above procedures, and a control experiment of photo-fluorination of F-ND catalysts in the absence of oxygen is also conducted in a similar procedure, except for completely removing the air before irradiation by a vacuum pump.

### Catalytic tests

Catalytic dehydrogenation of EB was carried out in a fixed-bed quartz reactor under atmospheric pressure. Typically, 20 mg catalysts were fixed between two quartz wool plugs and preheated to 400 °C in N_2_. Then the reactant (470 nmol/mL EB with N_2_ balance, O_2_$:$EB = 3:1, total flow rate of 10 mL/min, N_2_ as balance) was fed to the reactor. The reaction products were analyzed by Agilent 7890B gas chromatograph equipped with a HP-5 capillary column (length: 30 m) connected to a flame ionization detector (FID) for the hydrocarbons, and a Porapak Q packed column (length: 2 m) connected to a thermal conductivity detector (TCD) for the permanent gases. The temperature programme involved heating at 15 °C/min from 50 to 150 °C.

### Quantitative analysis of ketonic C=O groups

The method of quantitating the ketonic C=O groups was based on the hydrochloride hydroxylamine^34^.

1 $${{{{{\rm{RCOR}}}}}}\hbox{'}+{{{{{{\rm{H}}}}}}}_{2}{{{{{\rm{NOH}}}}}}\cdot {{{{{\rm{HCl}}}}}}\to {{{{{\rm{RR}}}}}}\hbox{'}{{{{{\rm{C}}}}}}={{{{{\rm{NOH}}}}}}+{{{{{{\rm{H}}}}}}}_{2}{{{{{\rm{O}}}}}}+{{{{{\rm{HCl}}}}}}$$

2 $${{{{{\rm{HCl}}}}}}+{{{{{\rm{NaOH}}}}}}={{{{{\rm{NaCl}}}}}}+{{{{{{\rm{H}}}}}}}_{2}{{{{{\rm{O}}}}}}$$

A solution of 3 × 10^−3^ M of bromophenol blue in ethanol was prepared. 1 mL of the bromophenol blue solution, 0.5 g hydroxylammonium chloride, and 25 mL ethanol were added into a 50 mL round-bottomed flask with 100 mg of catalysts (the fresh solution was bright yellow). The above solution was titrated with 0.05 M NaOH until it appeared yellowish green. After the titration, the solution was stirred and heated to 100 °C under a refluxing condition for 3 h, and allowed to cool to room temperature. Then, titrated the solution with 0.05 M NaOH until it appeared yellowish-green again and recorded the volume (*V*_*1*_). The blank experiment was carried out under the same conditions, and the consumption of 0.05 M NaOH was recorded as *V*_*0*_. Finally, the amount of substance for C=O was calculated as follows:

3 $$V={V}_{1}-{V}_{0}$$

4 $$n=c\times V$$

Where *n* is the amount of substance for C=O groups, *c* is the concentration of the NaOH standard solution, and *V* is the volume of the NaOH standard solution used for the titration, excluding the consumption of the blank experiment.

### Dehydrogenation of 1,2,3,4-tetrahydroquinoline (THQ) to quinolone

The dehydrogenation of THQ reaction was carried out in a 48 mL thick-walled pressure flask with magnetic stirring. 0.3 mmol of THQ solution, 10 mg catalyst, and 3.0 mL of isopropanol solution were added into the reactor and the reaction was performed for 5 h at the temperature of 40 °C. After cooling to room temperature, the liquid phase component was characterized with a gas chromatograph-mass spectrometer (GC-MS).

### Characterization

Powder XRD patterns were collected on Bruker D8 Advance diffractometer with Cu-K1 radiation (λ = 1.5406 Å). FT-IR spectra were performed on a Thermo Nicolet Nexus 670 FTIR spectrometer with KBr as the diluents. TEM and energy dispersive spectroscopy (EDS) mapping were obtained using an FEI Tencai 20 instrument or Thermo Fisher Scientific TEM (Themis Z, 300 kV). XPS measurements were performed on an ESCALAB 250 (Thermo Scientific, USA) by using a monochromatized Al Kα line source (200 W). The ^19^F NMR experiments were performed on a Bruker AVANCE III 500 MAS VTN 4 mm probe (solid). N_2_ adsorption−desorption measurements were performed on Micromeritics ASAP2020 equipment. The scanning emission microscope (SEM) measurements were carried out by using Hitachi New Generation SU8010. Raman spectroscopic measurements were performed with a Renishaw inVia Raman microscope (Invia Reflex) with a 325 nm excitation source at room temperature. TGA was performed on STA 449 F3 Jupiter (NETZSCH Co.). Gas chromatography-mass spectra (GC-MS) were carried out in Thermo Trace 1300 gas chromatograph-mass spectrometer and a TR-5MS column (0.25 mm × 30 m, Film: 0.25 μm).

### Details of the calculations

All the spin-polarized calculations were carried out by the Vienna Ab initio Simulation Package (VASP)^38–40^ with the gradient-corrected Perdew−Burke−Ernzerhof functional^41^. For valence electrons, a plane-wave basis set was used with a cutoff energy of 400 eV, and the ionic cores were described with the projector augmented-wave method^42^. The periodicity of nanodiamond systems (*a =* 10.1 Å, *b* = 10.1 Å, and *c* = 31.7 Å) were modelled with eight atomic layers containing a 2 × 2 unit cell of diamond (111) covered by a 4 × 4 unit cell of graphene. A vacuum space larger than 15 Å was employed between periodic slabs to avoid the artificial interactions along the *z*-direction. 2 × 2 × 1 Monkhorst−Pack k-point grids were adopted to sample the Brillouin zone^43^, which was tested to be converged. During geometrical optimization, the atoms in the bottom six atomic layers were fixed while other atoms and the adsorbates were fully relaxed until the force acting on each atom was less than 0.03 eV/Å, and the convergence criteria for the energy was set as 10^−4^ eV. The climbing image-nudged elastic band (CI-NEB) approach^44,45^ was employed to simulate the reaction energy barrier, the barrier was calculated as the energy difference between the transition state and the initial state. The reaction energy *ΔE* was calculated as the energy difference between the final state and the initial state.

## Supplementary information

Supplementary Information
