# Supplementary material for: Photo-fluorination of nanodiamonds catalyzing oxidative dehydrogenation reaction of ethylbenzene
Source: Nat Commun. 2021 Nov 11;12:6542. doi: 10.1038/s41467-021-26891-8 (PMC8586349; doi:10.1038/s41467-021-26891-8)
Supplement: Supplementary file 1 — Supplementary Information [file 41467_2021_26891_MOESM1_ESM.pdf]

Supplementary Information for

Photo-fluorination of nanodiamonds catalyzing oxidative dehydrogenation reaction of ethylbenzene

Zhishan Luo[1,2], Qiang Wan[1], Zhiyang Yu[1], Sen Lin[1], Zailai Xie[1] and Xinchen Wang*[1,2]*

1. State Key Laboratory of Photocatalysis on Energy and Environment, College of Chemistry, Fuzhou University, Fuzhou Fujian 350108, P. R. China.
2. College of Chemical Engineering, Fuzhou University, Fuzhou Fujian 350108, P. R. China.

*To whom correspondence should be addressed.

Email: zlxie@fzu.edu.cn; xcwang@fzu.edu.cn;

Website: http://wanglab.fzu.edu.cn

Supplementary Figures

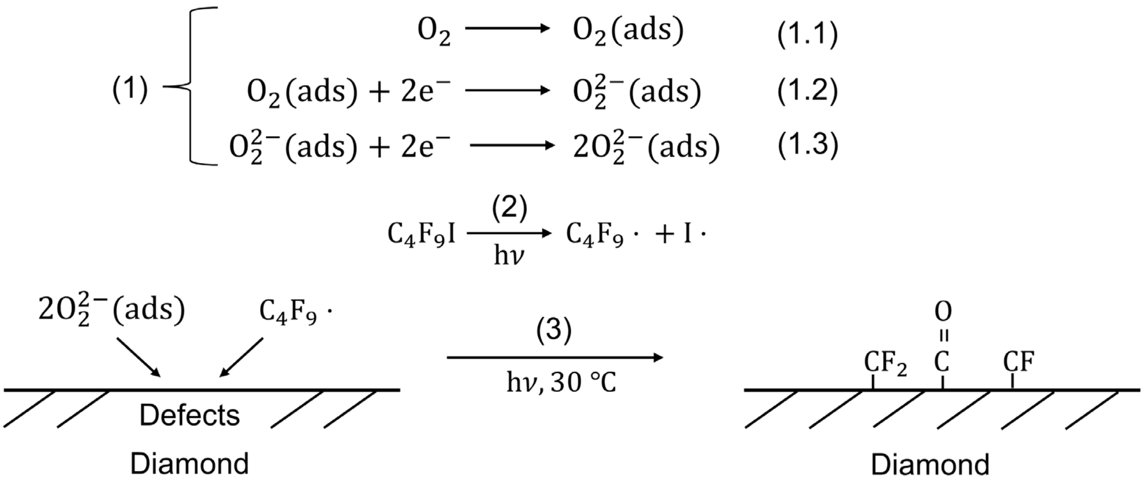

Supplementary Figure 1. A possible mechanism for the photo-fluorination of ND catalysts.
(**1.1**) Oxygen molecules are adsorbed on the defects of ND catalysts.
(**1.2, 1.3**) Molecular oxygen interacts with free electrons at the defect site to form atomic oxygen. Steps (**1.1**) to (**1.3**) designate the activation of molecular oxygen into an oxidizing atomic species[1].
(**2**) To generate free radicals of $C_4F_9\cdot$ by photolyzing C_4F_9I molecules[2].
(**3**) Formation of C-F, CF_2, and C=O bonds through oxidation and fluorination structural defects of ND catalysts.

Note that: The surface of nanodiamond (ND) is covered by a lot of structural defects due to it is mostly synthesized by the detonation technique[3]. We speculate that O_2 and the solution of C_4H_9I are easily adsorbed at the defect sites of ND catalysts. The mechanism of the photo-induced fluorination of ND catalysts can be divided into three steps:
(1) Designate the activation of molecular oxygen into an oxidizing atomic species.
(2) To generate free radicals of $C_4F_9\cdot$ by photolyzing C_4F_9I molecules.
(3) Formation of CF, CF_2, and C=O bonds through oxidation and fluorination structural defects of ND catalysts.

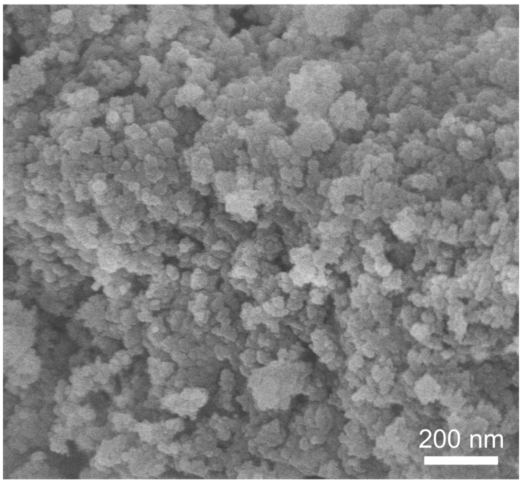

Supplementary Figure 2. SEM image of ND catalysts.

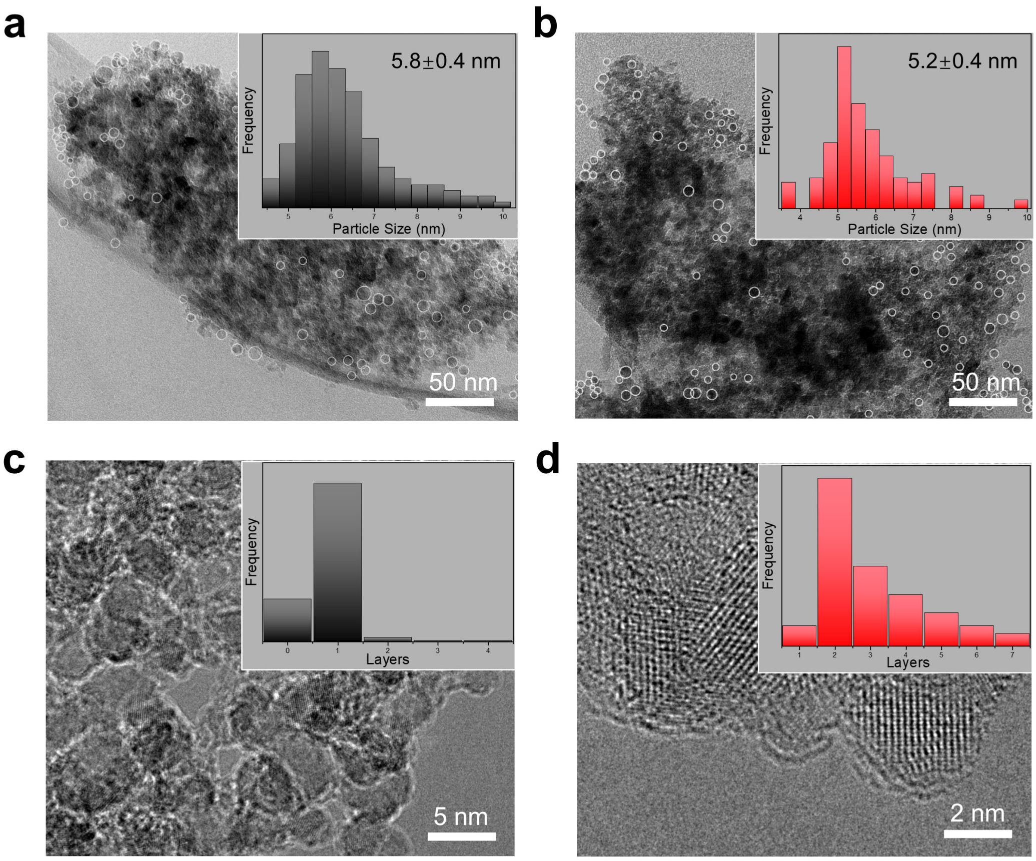

Supplementary Figure 3. TEM and HR-TEM images of (**a**, **c**) ND and (**b**, **d**) F-ND catalysts. Insets: the statistics histogram of particle size or graphitic layers.

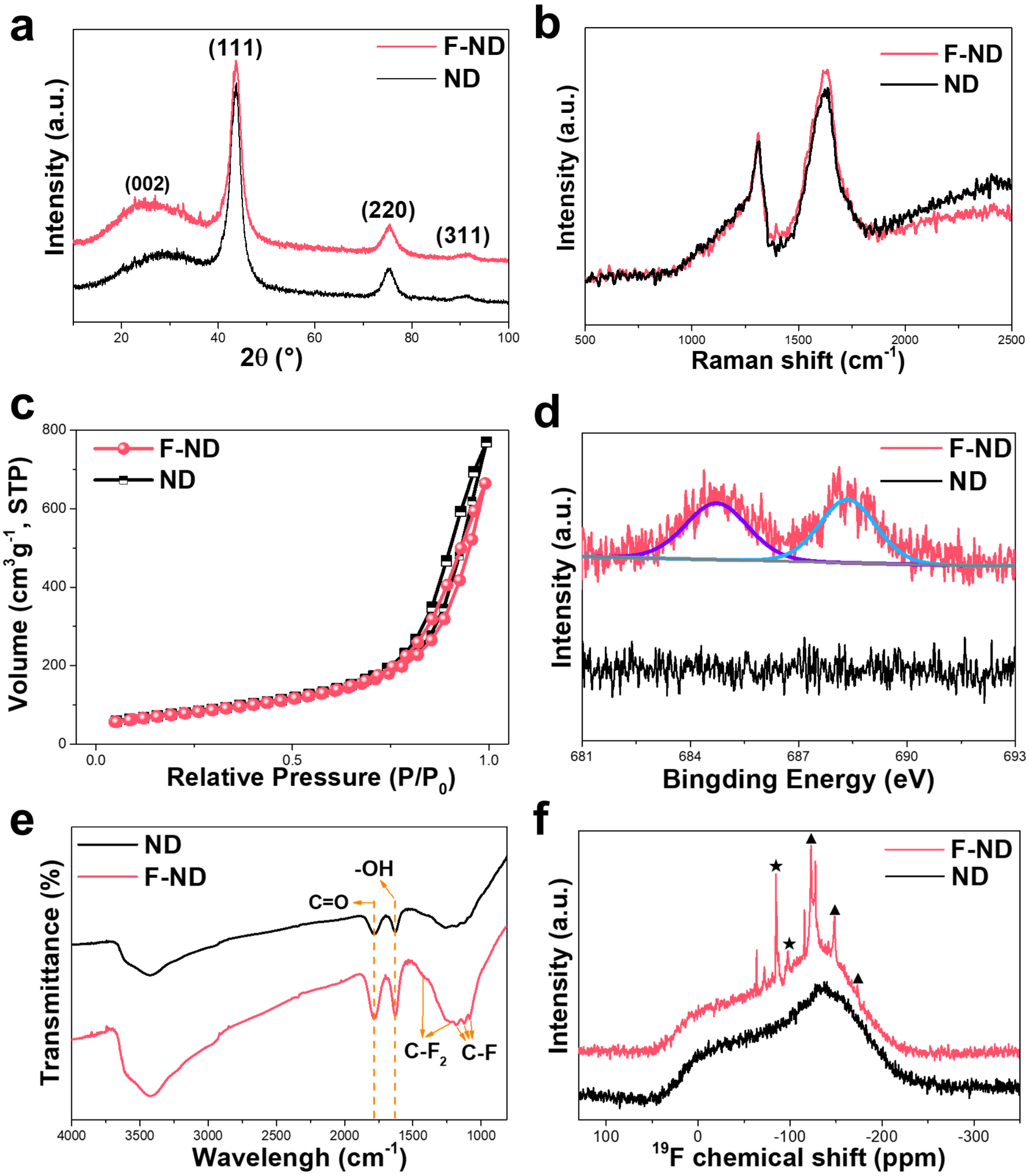

Supplementary Figure 4. (**a**) XRD patterns, (**b**) Raman spectra, (**c**) N_2 adsorption-desorption isotherms, (**d**) high-resolution XPS spectra of F 1s, (**e**) FT-IR spectra, and (**f**) ^{19}F solid-state spectra for ND and F-ND catalysts. Star, CF, triangle, CF_2. (Note that XPS data of F-ND catalysts in **d** are derived from Fig. 3b for the convenient comparison).

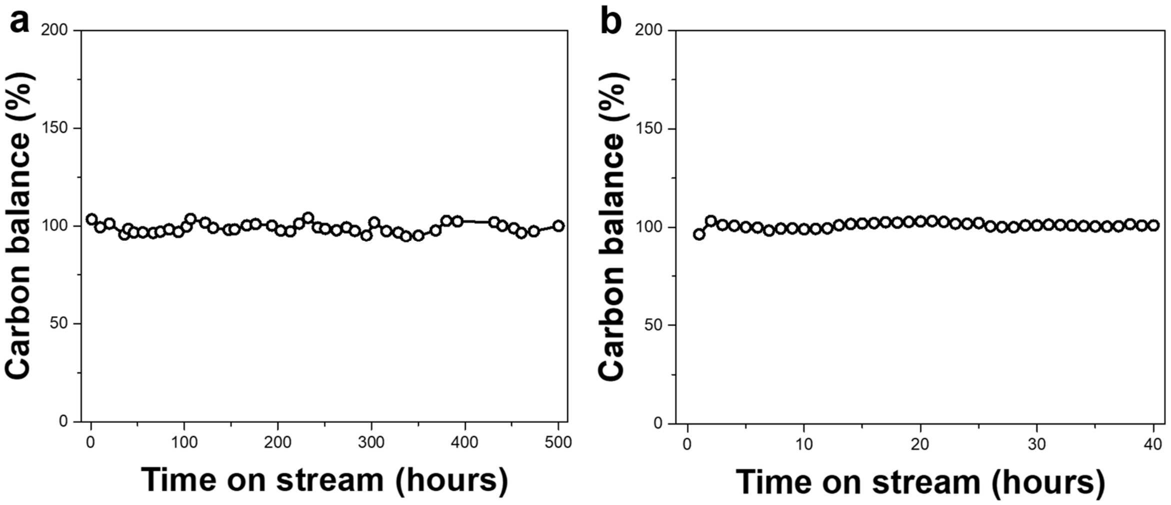

Supplementary Figure 5. Carbon balance during the catalytic test of various carbon-based catalysts for ODH reactions of EB to ST in (**a**): Fig. 2b, and (**b**): Fig. 2c.

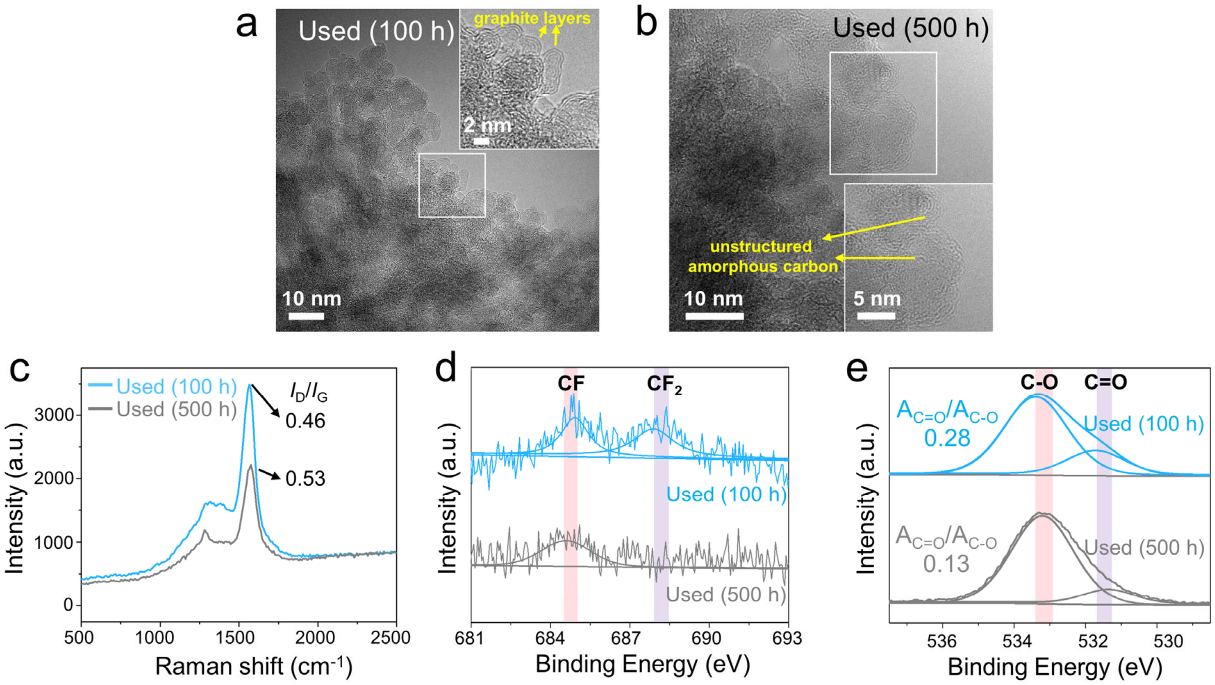

Supplementary Figure 6. (**a-b**) TEM images, (**c**) Raman spectra and high-resolution XPS of (**d**) F 1s and (**e**) O1s for the F-ND catalysts before and after the stability test of ODH reactions.

Note that: TEM images of the used F-ND catalysts after 100 and 500 hours test are listed in Supplementary Fig. 6a and b, respectively. Compared with the F-ND catalysts used for 100 hours, an obviously unstructured amorphous carbon was obtained after a 500-hour test, indicating the amorphous carbon on the surface of F-ND catalysts increased after 500 hours of measurement. This is further confirmed by the Raman spectra with the I_D/I_G increased from 0.46 to 0.53 (Supplementary Fig. 6c), and the formation of such amorphous carbon in the surface of F-ND catalysts will cause a partial decrease in catalytic performance due to it may cover the active sites[4]. Subsequently, we also tested the XPS of F 1s and O 1s for F-ND catalysts after the 100 and 500 hours test, respectively. The bond of CF_2 was disappeared for F-ND catalysts after a 500-hour test (Supplementary Fig. 6d), and the area of the peak ratio of C=O groups and C-O groups ($A_{C=O}/A_{C-O}$) was calculated as 0.28 and 0.13 (Supplementary Fig. 6e) for F-ND catalysts used for 100 and 500 hours test, respectively. These results show that the active sites of C=O groups on the surface of F-ND catalysts decrease after a long-term experiment. Therefore, we speculate that the cause of the deactivation is due to the formation of amorphous carbon on the surface covering the active sites and the reduction of the content of active sites during the reaction. The content of ketone groups, CF_2, amorphous carbon, etc. are kinetic and change with reaction time-on-stream.

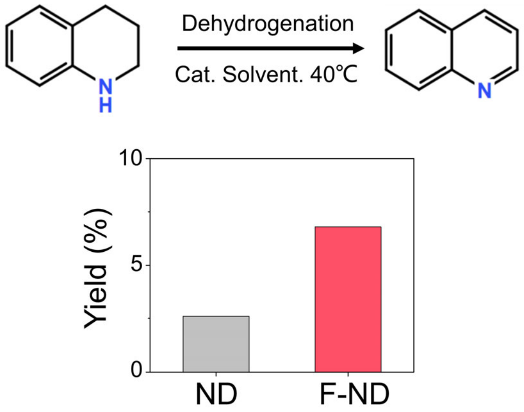

Supplementary Figure 7. The catalytic efficiency of ND and F-ND catalysts for the dehydrogenation of 1,2,3,4-tetrahydroquinoline (THQ) reactions. Reaction conditions: 0.3 mmol of THQ solution, 10 mg catalyst, and 3.0 mL of isopropanol solution at 40 °C for 5 h. The yield was determined by GC-MS.

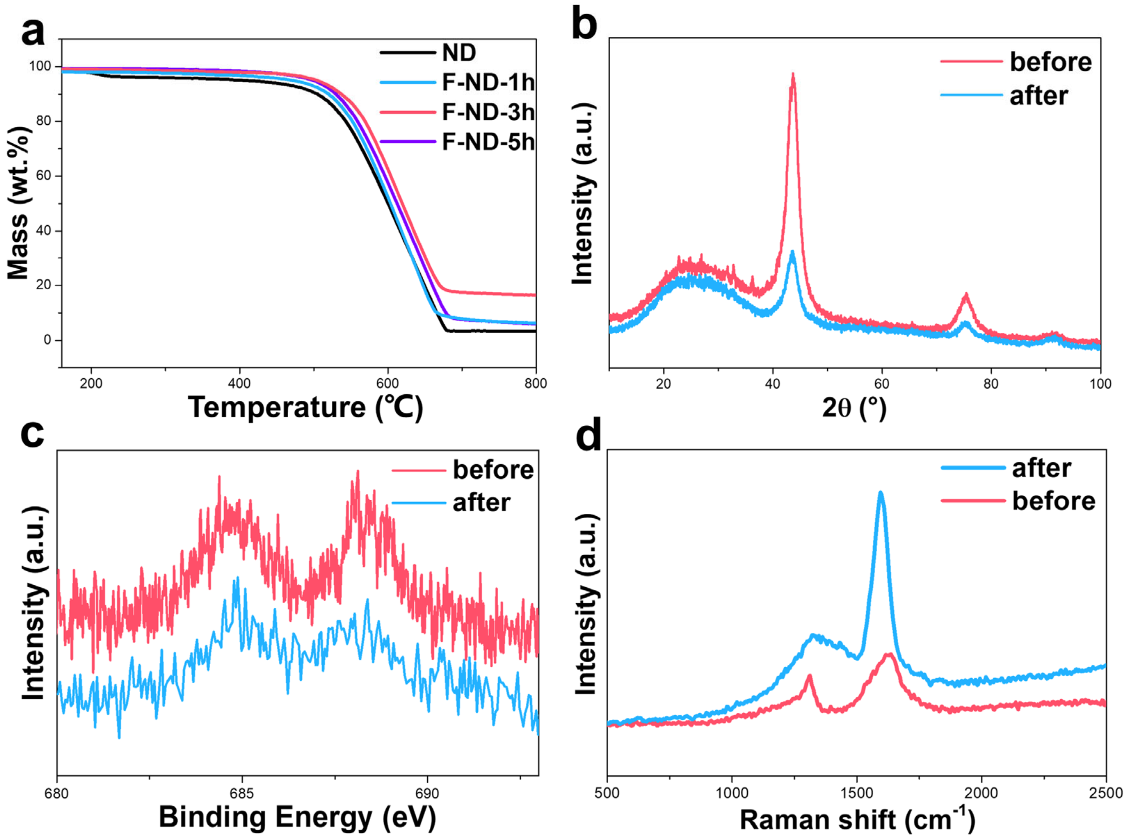

Supplementary Figure 8. (**a**) The thermogravimetric analysis (TGA) experimental curves of ND and F-ND catalysts in the air condition. The spectra of F-ND catalysts before and after the ODH reaction (used 100 h) for (**b**) XRD, (**c**) XPS, and (**d**) Raman spectra.

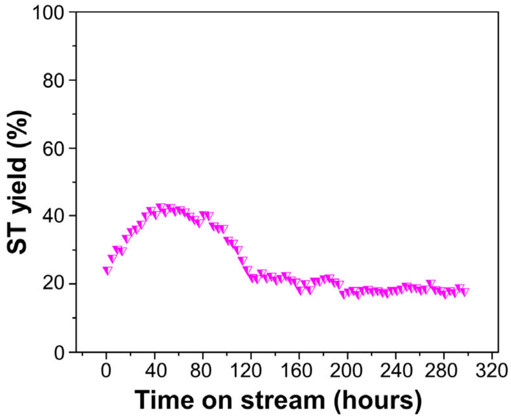

Supplementary Figure 9. Stability of NC catalysts for ODH reactions over a 300-hour test. Reaction condition: 20 mg catalysts, 470 nmol mL^{-1} EB with N_2 balance, O_2: EB=3:1, total flow rate = 10 mL min^{-1}, T= 400 ℃. NC: nanocarbons.

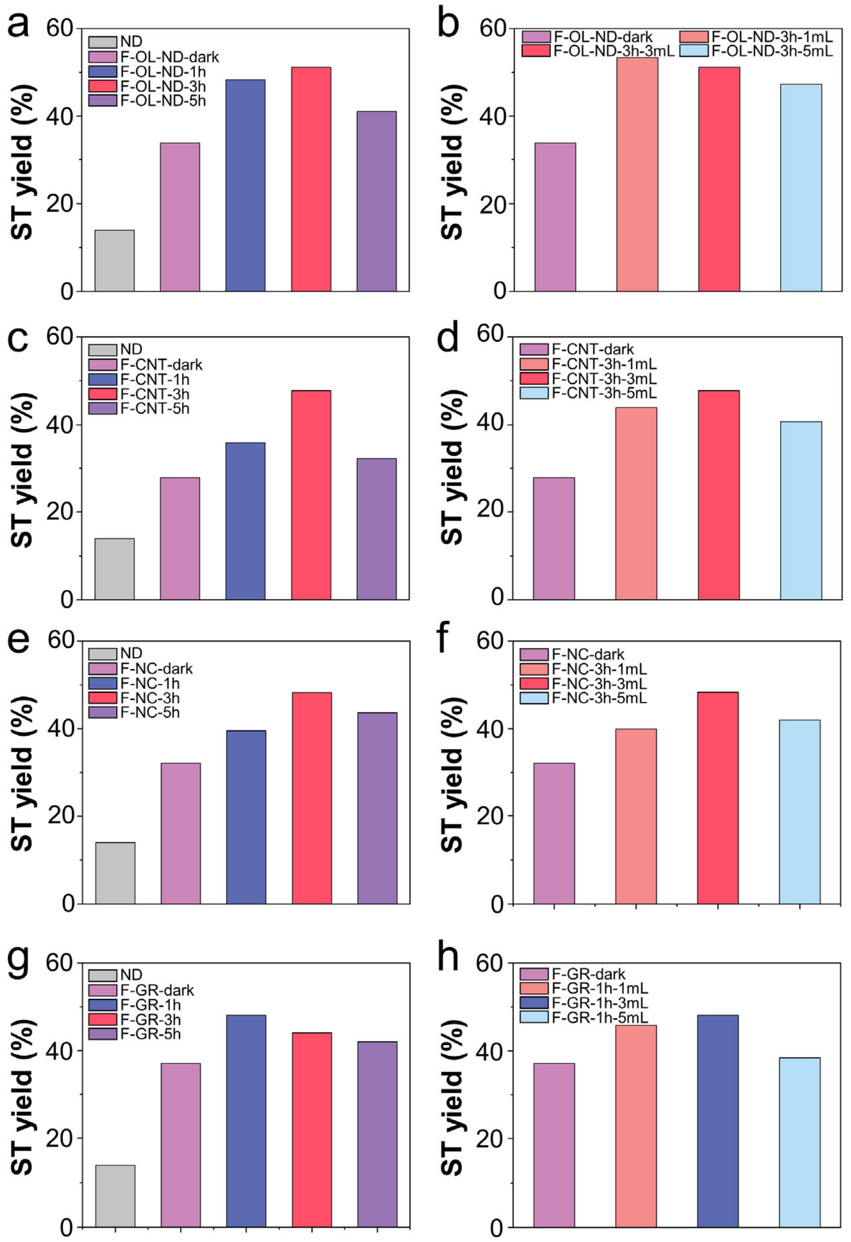

Supplementary Figure 10. The catalytic performance of ODH reactions for the (**a-b**) OL-ND, (**c-d**) CNT, (**e-f**) NC, and (**g-h**) GR catalysts under different photo-fluorination times and different content of C_4F_9I solution. Reaction conditions: 20 mg catalysts, 470 nmol mL^{-1} EB with N_2 balance, O_2 : EB=3:1, total flow rate = 10 mL min^{-1}, T=400 °C. It is worth noting that the optimized photo-fluorination time solution for F-OL-ND, F-CNT, F-NC, and F-GR catalysts is 3, 3, 3, and 1h, respectively, and the optimized content of the C_4F_9I solution for F-OL-ND, F-CNT, F-NC, and F-GR catalysts is 1, 3, 3 and 3 mL, respectively.

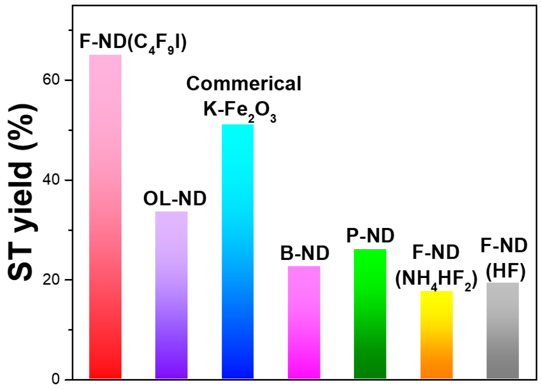

Supplementary Figure 11. The catalytic performance of various element-modified ND catalysts and commercial catalysts for ODH reactions.

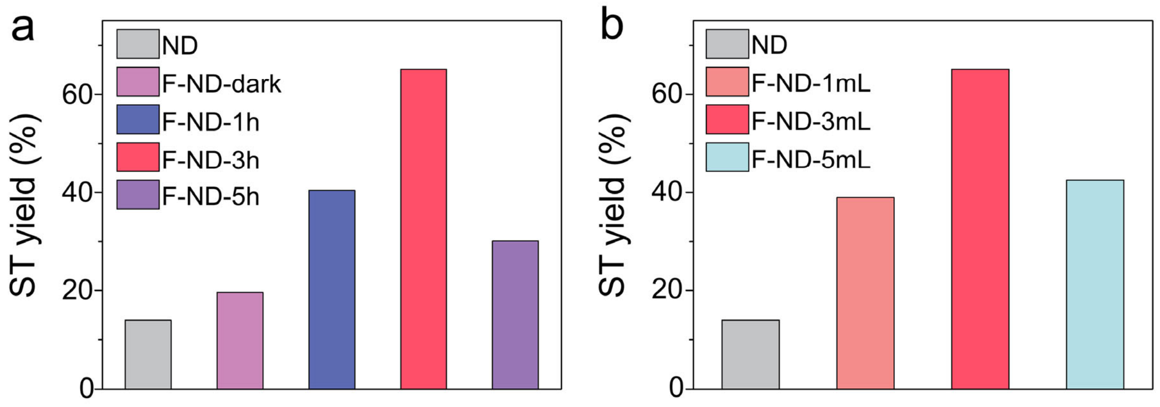

Supplementary Figure 12. The catalytic performance of ODH reactions for the F-ND catalysts under (**a**) different photo-fluorination time and (**b**) different content of C_4F_9I solution. Reaction conditions: 20 mg catalysts, 470 nmol mL^{-1} EB with N_2 balance, O_2 : EB=3:1, total flow rate = 10 mL min^{-1}, T=400 °C.

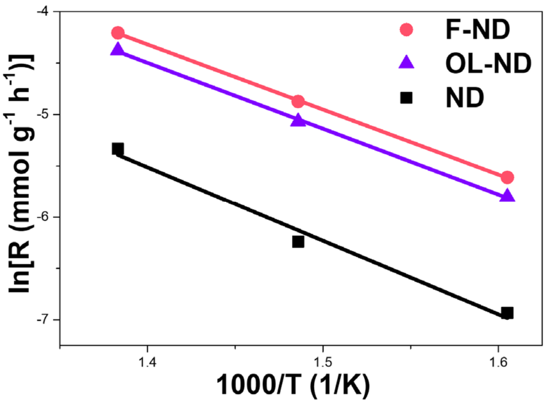

Supplementary Figure 13. Temperature dependencies of intrinsic activities for ODH reactions over ND, OL-ND, F-ND catalysts in a differential reactor. (470 nmol mL^{-1} EB with N_2 balance, O_2 : EB=3:1, total flow rate = 10 mL/min, 350-450 ℃).

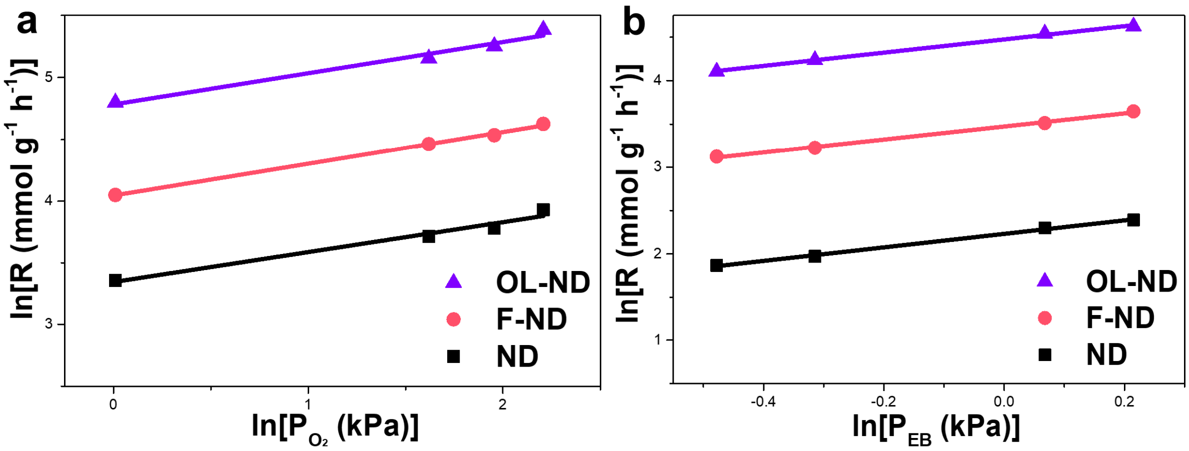

Supplementary Figure 14. (**a**) Influence of O_2 (1.0-9.1 kPa O_2) and (**b**) EB (0.62-1.24 kPa EB) partial pressures on the rate of styrene formation over ND, OL-ND, and F-ND catalysts (N_2 as balance, 400 °C).

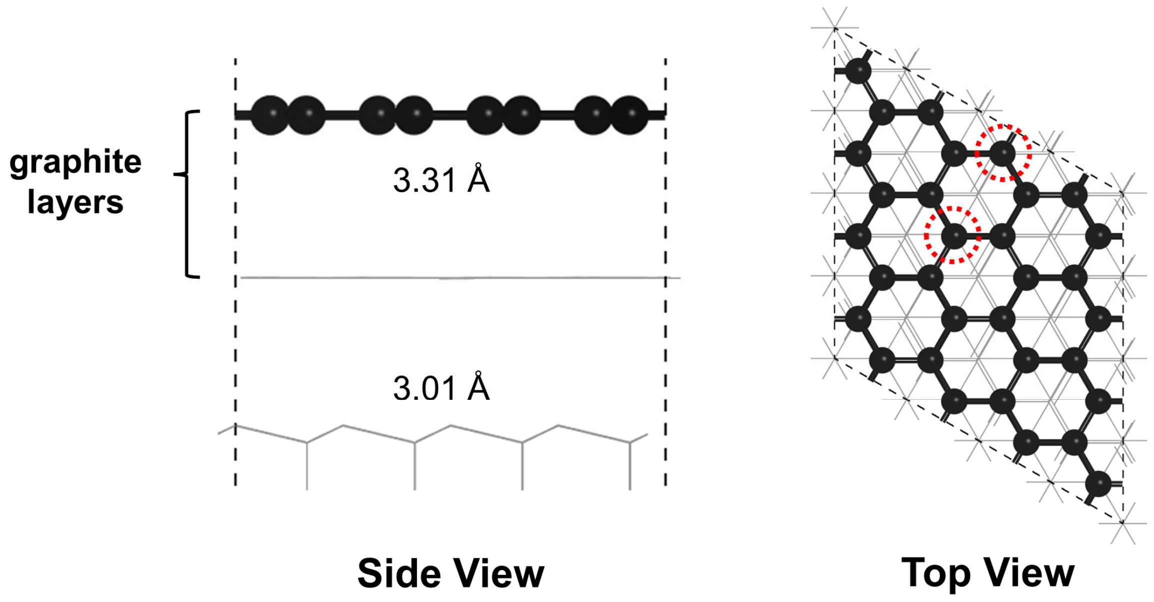

Supplementary Figure 15. Side and top views of nanodiamond covered with two monolayers of graphene. The surface C atoms marked by red dash circles at the outmost monolayer are removed to construct vacancies. Color scheme: C in the outmost graphene surface, black; other C atoms, grey.

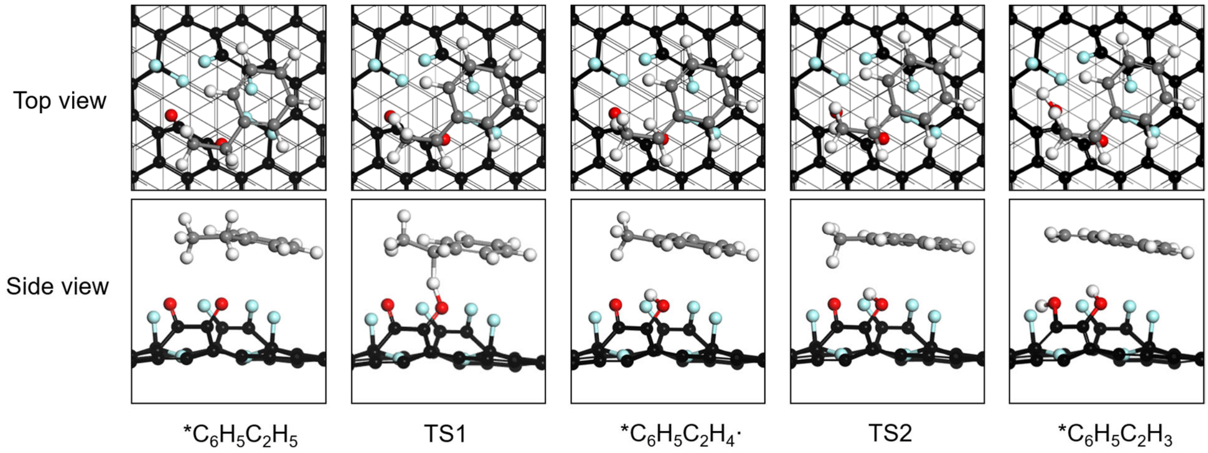

Supplementary Figure 16. Corresponding structures along the dehydrogenation of EB over F-ND catalysts without an F located at an adjacent carbon atom to C=O. TS: transition state. Color scheme: C in the outmost graphene surface, black; other C atoms, grey; O, red; F, azure.

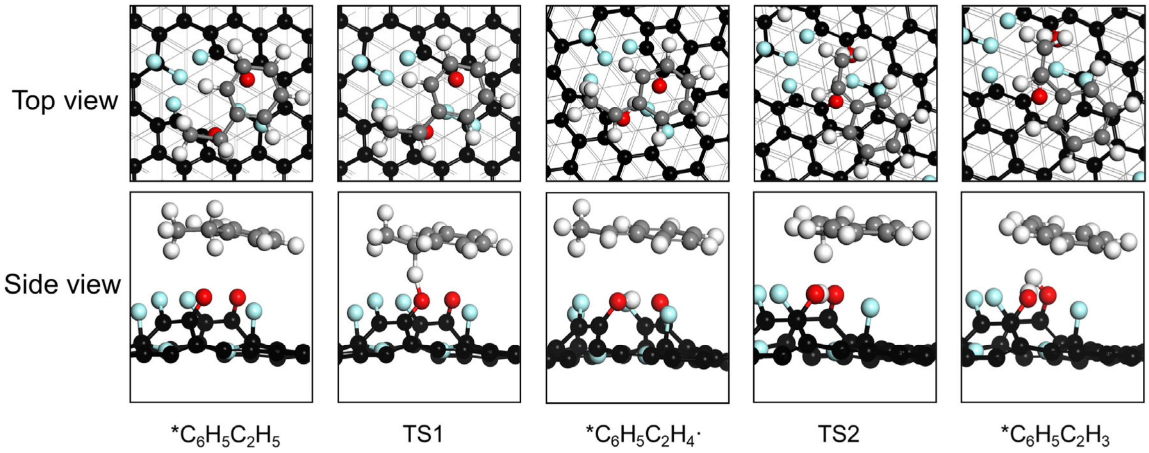

Supplementary Figure 17. Corresponding structures along the dehydrogenation of EB over F-ND catalysts with an F located at an adjacent carbon atom to C=O. TS: transition state. Color scheme: C in the outmost graphene surface, black; other C atoms, grey; O, red; F, azure.

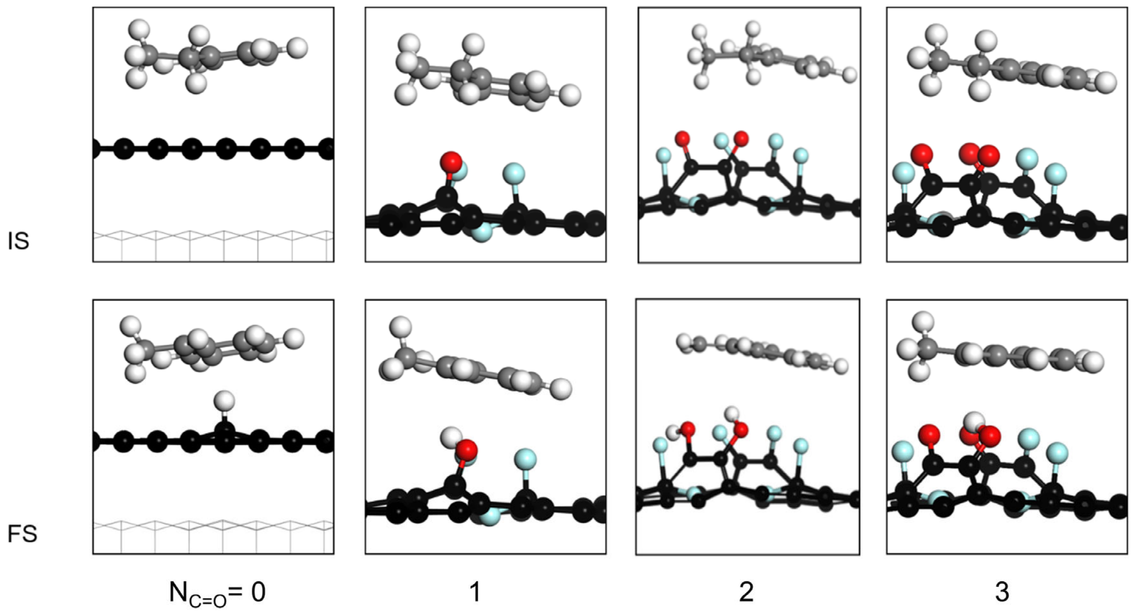

Supplementary Figure 18. Configurations of the initial and final states of the first dehydrogenation of EB on catalysts with the different number of C=O groups ($N_{C=O}$ =0, 1, 2, and 3). Color scheme: C in the outmost graphene surface, black; other C atoms, grey; O, red; F, azure.

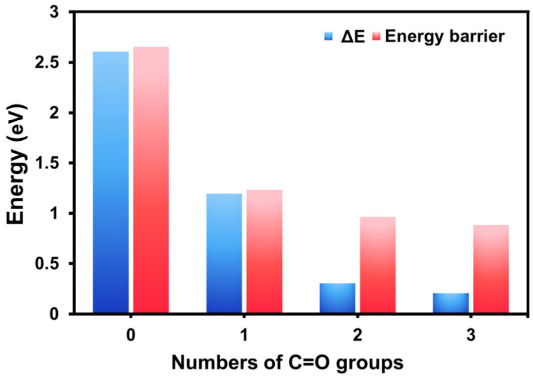

Supplementary Figure 19. Calculated reaction energy and barrier of the first dehydrogenation of EB on catalysts with different numbers of C=O groups. The corresponding structures were displayed in Supplementary Fig. 18.

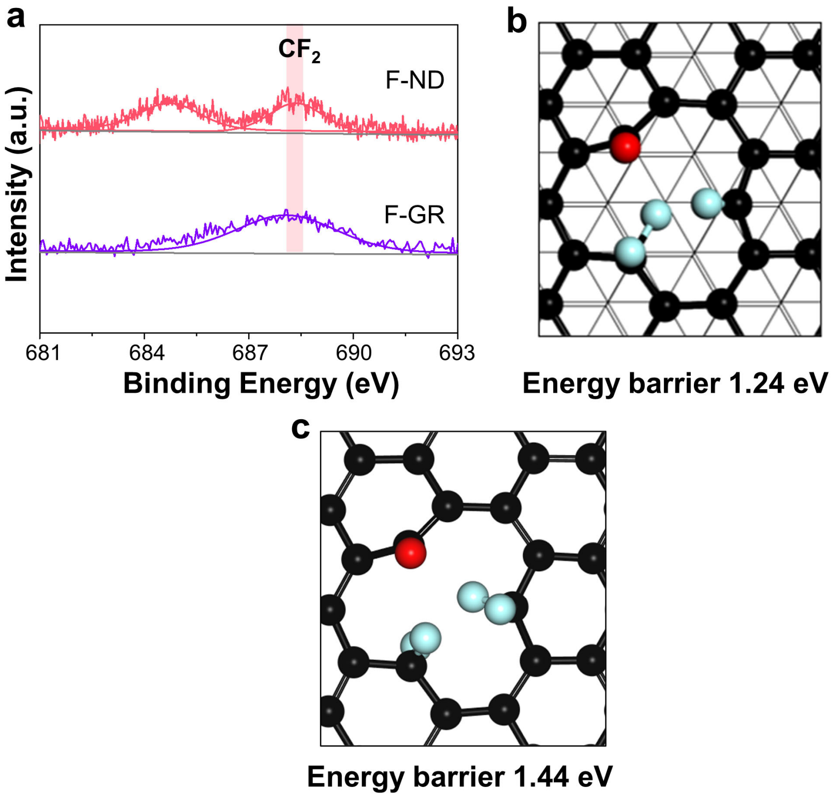

Supplementary Figure 20. (**a**) High-resolution XPS spectra of F 1s for F-ND and F-GR catalysts. The calculated energy barrier of ODH reactions for (**b**) F-ND catalysts (bonds: C=O, CF, and CF_2), (**c**) F-GR catalysts (bonds: C=O and CF_2). Note that the calculated structure of F-GR catalysts is constructed by removing the nanodiamonds from the calculated structure of F-ND catalysts (Supplementary Fig. 18, $N_{C=O}$=1). In F-GR catalysts, only CF_2 bonds are available. Color scheme: C in the outmost graphene surface, black; other C atoms, grey; O, red; F, azure. (Note that XPS data of F-ND catalysts in **a** are derived from Fig. 3b for convenient comparison).

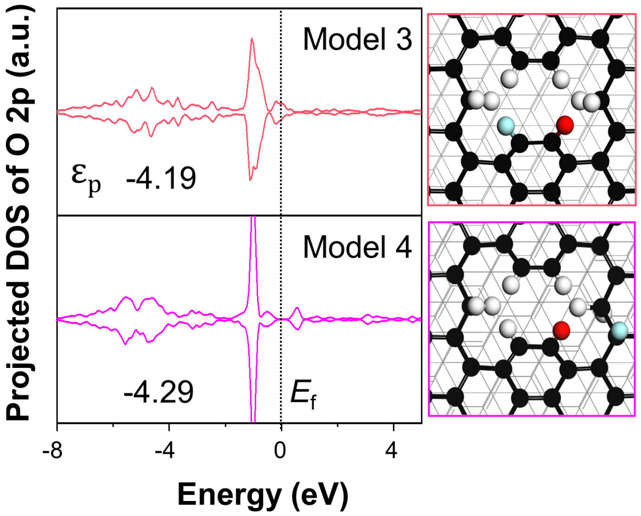

Supplementary Figure 21. The calculated projected density of states (PDOSs) of O_{2p} states for Model 3 (upper panel) with C-F bonds adjacent to C=O bonds, and Model 4 (lower panel) with bonds of C-F and C=O separated by three C-C bonds, E_F: Fermi level, ε_p: *p*-band center. Color scheme: C in the outmost graphene surface, black; other C atoms, grey; O, red; F, azure.

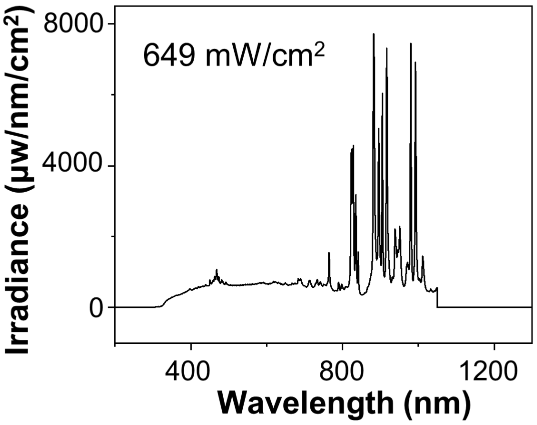

Supplementary Figure 22. The spectrum of the irradiation power density for the 300 W xenon lamp.

Supplementary Tables

Supplementary Table 1. Kinetic parameters for ODH of ethylbenzene over ND, OL-ND, and F-ND catalysts, respectively.

Catalysts	ODH rate at 400 °C [mmolg^{-1} h^{-1}]	Activation energies (kJ mol^{-1})	Reaction orders	
			EB	O_2
F-ND	0.76	52.5	0.75	0.24
OL-ND	0.62	53.2	0.76	0.25
ND	0.19	59.6	0.78	0.26

Supplementary Table 2. The data of DH and ODH of EB to ST for various catalysts.

Catalysts	Mass (mg)	Condition	S_{BET} [a] (m^2g^{-1})	Conversion (selectivity) (%)	Ref.
F-ND	20	ODH, O_2/EB=3:1, 400 °C	275	70 (92)	This work
Al_2O_3@C	50	ODH, O_2/EB=1:5, 450 °C	128	17 (95)	Ref [5]
BCN	30	ODH, O_2/EB=4:1, 500 °C	1450	54 (89)	Ref [6]
ND	50	ODH, O_2/EB=1:5, 450 °C	Not given	40 (92)	Ref [7]
CeO_2	200	DH, CO_2/EB=100:1, 450 °C	202	29 (80)	Ref [8]
V/AC [b]	100	DH, CO_2/EB=70:1, 550 °C	774	67.1 (80.8)	Ref [9]
VO_x/SiO_2	100	DH, CO_2/EB=20:1, 550 °C	83	50.5 (96.8)	Ref [10]
V/MgO	100	DH, CO_2/EB=45:1, 600 °C	144	81.9 (90.1)	Ref [11]
V/MCF [c]	80	DH, CO_2/EB=20:1, 550 °C	420	70.7 (98.0)	Ref [12]
K_2O/TiO_2-ZrO_2	1000	DH, CO_2/EB=5:1, 550 °C	96	71.9 (99.6)	Ref [13]
SnO_2-ZrO_2	1000	DH, CO_2/EB=6:1, 600 °C	Not given	61.1 (97.6)	Ref [14]
K/TiO_2-ZrO_2	1000	DH, CO_2/EB=5:1, 600 °C	256	75 (95)	Ref [15]
Na/TiO_2-ZrO_2			199	72 (97)	
Ce-Mn oxides/CNTs [d]	50	DH, CO_2/EB (15 $mL\cdot min^{-1}$), 550 °C	Not given	50.9 (99.2)	Ref [16]
$FeAl_2O_4$/SBA-15	100	DH, CO_2/EB=30:1, 550 °C	107	20.1 (98.7)	Ref [17]
$NiAl_2O_4$/SBA-15			96	12 (56)	
$CuAl_2O_4$/SBA-15			88	8.5 (99.8)	
$MgAl_2O_4$/SBA-15			118	10 (100)	

$MgFe_{0.1}Al_{1.9}O_4$	1000	DH, CO_2/EB=5:1, 580 °C	106	40.9 (95.3)	Ref [18]
$Fe_2O_3/MgAl_2O_4$			112	50 (95.7)	
Fe_2O_3-MgO/γ-Al_2O_3			202	24.5 (97.1)	
$Ce_{0.8}Fe_{0.2}O_2$	725	DH, CO_2/EB=20:1, 550 °C	63.9	33 (95.8)	Ref [19]
Fe_2O_3/Al_2O_3-ZrO_2	400	DH, CO_2/EB=10:1, 550 °C	200	39.5 (96.5)	Ref [20]
ND/CNT-SiC	500	DH, 10 mL·min^{-1} He with 2.6 % EB, 550 °C	Not given	19.4 (98.3)	Ref [21]
Co/CNTs	3000	ODH, air/EB=600:1, 700 °C	Not given	93.5 (80.6)	Ref [22]
CNFs [e)]	60	ODH, O_2/EB=2.6:1, 475 °C	99	21.5 (80.8)	Ref [23]
rPGO [f)]	50	ODH, O_2/EB=0.5:1, 400 °C	2613	50 (95)	Ref [24]
ND	50	DH, 10 mL·min^{-1} He with 2.8 % EB, 550 °C	Not given	21.1 (97.3)	Ref [25]
Onion-like carbon	40	ODH, O_2/EB=1:1, 517 °C	Not given	90 (72)	Ref [26]
Popcorn carbon	50	ODH, O_2/EB=1:1, 400 °C	1417	48 (86)	Ref [27]
CNTs	200	ODH, O_2/EB=1:1, 450 °C	271	28 (68)	Ref [28]

[a)]Specific surface area; [b)]Activated Carbon; [c)]Mesocellular silica foam; [d)]Carbon nanotubes; [e)]Nanofibers; [f)]Reduced porous graphene oxide;

Supplementary Table 3. The content of elemental carbon, oxygen, and fluorine from XPS experiments of ND and F-ND catalysts.

Catalysts	Carbon (at %)	Oxygen (at %)	Fluorine (at %)	$A_{C=O}$ / A_{C-O}[a)]
ND	90.2	9.8	-	0.11
F-ND-1h	88.9	10.7	0.2	0.28
F-ND-3h	87.7	11.8	0.5	0.42
F-ND-5h	83.8	13.9	2.3	0.20

[a)]The area of peak ratio of C=O groups and C-O groups

Supplementary Table 4. The data of titration experiments for ND and F-ND catalysts.

Catalysts	Mass (mg)	0.05 M NaOH (**V** mL) (standard solution）	$n_{C=O}$[b)] (μmol)		EB conversion[c)] (%)	ST yield[d)] (%)
ND	100	0.10[a)]	5.0	5.2[e)]	14.6	13.9
		0.10	5.0			
		0.11	5.5			
F-ND-1h	100	0.44	22.0	23.3	42.1	40.4
		0.48	24.0			
		0.48	24.0			
F-ND-3h	100	0.80	40.0	39.3	70.8	65.2
		0.80	40.0			
		0.76	38.0			
F-ND-5h	100	0.40	20.0	20.7	31.8	30.1
		0.44	22.0			
		0.40	20.0			

[a)]Titration 1 mL with 20 drops; [b)]Amount of substance for C=O groups; [c)]The conversion of ethylbenzene; [d)]The yield of styrene; [e)]Average of three titration experiments

Supplementary References

1. Ertl, G., Knözinger, H., Schüth, F. & Weitkamp, J. *Handbook of heterogeneous catalysis*, vol. 1. Wiley-VCH: Weinheim, 2008.
2. Smentkowski, V. S. & Yates, J. T. Fluorination of diamond surfaces by irradiation of perfluorinated alkyl iodides. *Science* **271**, 193-195 (1996).
3. Mochalin, V. N., Shenderova, O., Ho, D. & Gogotsi, Y. The properties and applications of nanodiamonds. *Nat Nanotechnol* **7**, 11-23 (2011).
4. Yan, P. *et al.* Surface chemistry of nanocarbon: Characterization strategies from the viewpoint of catalysis and energy conversion. *Carbon* **143**, 915-936 (2019).
5. Wang, J. *et al.* Few-layer sp^2 carbon supported on Al_2O_3 as hybrid structure for ethylbenzene oxidative dehydrogenation. *Catal. Today* **301**, 32-37 (2018).
6. Guo, F. *et al.* Carbon-doped BN nanosheets for the oxidative dehydrogenation of ethylbenzene. *Angew. Chem. Int. Ed.* **56**, 8231-8235 (2017).
7. Diao, J. *et al.* Selective and stable ethylbenzene dehydrogenation to styrene over nanodiamonds under oxygen-lean conditions. *ChemSusChem* **9**, 662-666 (2016).
8. Zhang, L. *et al.* Role of CO_2 as a soft oxidant for dehydrogenation of ethylbenzene to styrene over a high-surface-area ceria catalyst. *ACS Catal.* **5**, 6426-6435 (2015).
9. Sakurai, Y., Suzaki, T., Ikenaga, N.-o. & Suzuki, T. Dehydrogenation of ethylbenzene with an activated carbon-supported vanadium catalyst. *Appl. Catal. A: Gen.* **192**, 281-288 (2000).
10. Chen, S. *et al.* Promoting effect of carbon dioxide on the dehydrogenation of ethylbenzene over silica-supported vanadium catalysts. *Fuel* **109**, 43-48 (2013).
11. Sakurai, Y. *et al.* Dehydrogenation of ethylbenzene over vanadium oxide-loaded MgO catalyst: Promoting effect of carbon dioxide. *J. Catal.* **209**, 16-24 (2002).
12. Li, C. *et al.* Ethylbenzene dehydrogenation in the presence of CO_2 over MCF-supported vanadium oxide catalysts. *Chin. J. Catal.* **31**, 993-998 (2010).
13. Burri, D. R. *et al.* Selective conversion of ethylbenzene into styrene over K_2O/TiO_2-ZrO_2 catalysts: Unified effects of K_2O and CO_2. *J. Mol. Catal. A: Chem.* **269**, 58-63 (2007).
14. Burri, D. R. *et al.* Oxidative dehydrogenation of ethylbenzene to styrene with CO_2 over SnO_2–ZrO_2 mixed oxide nanocomposite catalysts. *Catal. Today* **131**, 173-178 (2008).
15. Burri, A., Jiang, N., Yahyaoui, K. & Park, S.-E. Ethylbenzene to styrene over alkali doped TiO_2-ZrO_2 with CO_2 as soft oxidant. *Appl. Catal. A: Gen.* **495**, 192-199 (2015).
16. Li, C., Sun, Y. & Zhang, A. Binary Ce–Mn oxides confined in carbon nanotubes as efficient catalysts for ethylbenzene dehydrogenation in the presence of carbon dioxide. *RSC Adv.* **5**, 36394-36403 (2015).
17. Batista, A. H. d. M. *et al.* Mesoporous MAl_2O_4 (M=Cu, Ni, Fe or Mg) spinels: Characterisation and application in the catalytic dehydrogenation of ethylbenzene in the presence of CO_2. *Appl. Catal. A: Gen.* **382**, 148-157 (2010).
18. Ji, M. *et al.* Dehydrogenation of ethylbenzene to styrene with CO_2 over iron oxide-based catalysts. *Catal. Today* **158**, 464-469 (2010).
19. Wang, Q., Li, X., Li, W. & Feng, J. Promoting effect of Fe in oxidative dehydrogenation of ethylbenzene to styrene with CO_2 (I) preparation and performance of $Ce_{1-x}Fe_xO_2$ catalyst. *Catal. Commun.* **50**, 21-24 (2014).
20. Zhang, S.-j., Li, W.-y. & Li, X.-h. Effect of preparation methods on the catalytic properties of Fe_2O_3/Al_2O_3-ZrO_2 for ethylbenzene dehydrogenation. *J. Fuel Chem. Technol.* **43**, 437-441 (2015).

21. Liu, H. *et al.* A nanodiamond/CNT-SiC monolith as a novel metal free catalyst for ethylbenzene direct dehydrogenation to styrene. *Chem. Commun.* **50**, 7810-7812 (2014).
22. Guo, X.-F., Kim, J.-H. & Kim, G.-J. Dehydrogenation of ethylbenzene to styrene on a direct synthesized Co, Ni/carbon nanotubes catalysts. *Catal. Today* **164**, 336-340 (2011).
23. Delgado, J. J. *et al.* Activation processes of highly ordered carbon nanofibers in the oxidative dehydrogenation of ethylbenzene. *Catal. Today* **186**, 93-98 (2012).
24. Diao, J. *et al.* Porous graphene-based material as an efficient metal free catalyst for the oxidative dehydrogenation of ethylbenzene to styrene. *Chem. Commun.* **51**, 3423-3425 (2015).
25. Zhang, J. *et al.* Surface chemistry and catalytic reactivity of a nanodiamond in the steam-free dehydrogenation of ethylbenzene. *Angew. Chem. Int. Ed.* **49**, 8640-8644 (2010).
26. Keller, N. *et al.* The catalytic use of onion-like carbon materials for styrene synthesis by oxidative dehydrogenation of ethylbenzene. *Angew. Chem. Int. Ed.* **41**, 1885-1888 (2002).
27. Wang, L. *et al.* Simple preparation of honeycomb-like macrostructured and microporous carbons with high performance in oxidative dehydrogenation of ethylbenzene. *Chem. Mater.* **19**, 2894-2897 (2007).
28. Pereira, M. F. R. *et al.* Catalytic activity of carbon nanotubes in the oxidative dehydrogenation of ethylbenzene. *Carbon* **42**, 2807-2813 (2004).
